# Stomatal Closure and Rise in ROS/NO of Arabidopsis Guard Cells by Tobacco Microbial Elicitors: Cryptogein and Harpin

**DOI:** 10.3389/fpls.2017.01096

**Published:** 2017-06-21

**Authors:** Gunja Gayatri, Srinivas Agurla, Kazuyuki Kuchitsu, Kondreddy Anil, Appa R. Podile, Agepati S. Raghavendra

**Affiliations:** ^1^Department of Plant Sciences, School of Life Sciences, University of HyderabadHyderabad, India; ^2^Department of Applied Biological Science, Tokyo University of ScienceChiba, Japan

**Keywords:** Arabidopsis, guard cells, innate immunity, microbial elicitors, nitric oxide, reactive oxygen species, signal transduction, stomatal closure

## Abstract

Plants use stomatal closure mediated by elicitors as the first step of the innate immune response to restrict the microbial entry. We present a comprehensive study of the effect of cryptogein and harpin, two elicitors from microbial pathogens of tobacco, on stomatal closure and guard cell signaling components in *Arabidopsis thaliana*, a model plant. Cryptogein as well as harpin induced stomatal closure, while elevating the levels of reactive oxygen species (ROS) and nitric oxide (NO) in the guard cells of *A. thaliana*. Kinetic studies with fluorescent dyes revealed that the rise in ROS levels preceded that of NO in guard cells, when treated with these two elicitors. The restriction of NO levels in guard cells, even by ROS modulators indicates the essentiality of ROS for NO production during elicitor-triggered stomatal closure. The signaling events during elicitor-induced stomatal closure appear to converge at NADPH oxidase and ROS production. Our results provide the first report on stomatal closure associated with rise in ROS/NO of guard cells by cryptogein and harpin in *A. thaliana*. Our results establish that *A. thaliana* can be used to study stomatal responses to the typical elicitors from microbial pathogens of other plants. The suitability of Arabidopsis opens up an excellent scope for further studies on signaling events leading to stomatal closure by microbial elicitors.

## Introduction

Stomata, the microscopic pores on the epidermis are the gateways for not only CO_2_ and H_2_O (Hetherington and Woodward, 2003) but also for microbes (Agurla et al., 2014). Stomatal closure therefore is essential to prevent pathogen entry into leaves and forms a part of their innate immune response (Zeng et al., 2010; Sawinski et al., 2013; Melotto et al., 2014). Production of elicitors has been considered as one of the mechanisms to induce stomatal closure in response to microbial pathogens. The elicitors (derived from either pathogens or from plants) induce several defense responses, besides stomatal closure, including ROS production, HR, cell death and production of antimicrobial secondary metabolites (Boller and Felix, 2009; Cui et al., 2009; Tsuda and Katagiri, 2010; Schwessinger and Ronald, 2012; Spoel and Dong, 2012; Newman et al., 2013). Compared to the extensive literature on elicitor effects on plant tissues, particularly cell cultures, the studies on stomatal closure by elicitors are limited.

The stomatal aperture is determined by the turgor status of guard cells and is mediated by the modulation of ion fluxes (Kim et al., 2010). The mechanism and signaling components of stomatal closure by ABA have been extensively studied in plants, such as *Arabidopsis thaliana, Pisum sativum*, and *Vicia faba*. ABA induced stomatal closure is invariably associated with marked increase in ROS, NO, and pH of guard cells (Gayatri et al., 2013; Agurla and Raghavendra, 2016). Further, several signaling components, are also involved in ABA-induced stomatal closure such as PYR/RCAR-PP2C-SnRK2 complexes, G-proteins, phospholipids, OST1, free Ca^2+^ and finally cation/anion channels (Hubbard et al., 2010; Raghavendra et al., 2010; Lee and Luan, 2012; Zhu et al., 2012; Murata et al., 2015). The transduction of both biotic and abiotic signals appears to share some common signaling components in guard cells, such as ROS and NO (Melotto et al., 2006; Srivastava et al., 2009; Khokon et al., 2010a,b; Zeng et al., 2010; Zhang et al., 2012; Agurla et al., 2014).

In view of the importance of ROS and NO as key signaling components during stomatal closure, extensive studies have been made on their sources. In guard cells ROS production can be mediated by enzymes such as NADPH oxidase, cell wall peroxidases, amine oxidases, and other flavin containing enzymes (Song et al., 2014). Most of these observations were made during stomatal closure by ABA, MeJA, methylglyoxal, and bicarbonate (Allan and Fluhr, 1997; Hoque et al., 2012). Very few studies were made on the RBOH dependency in elicitor mediated stomatal closure. Stomatal closure by flg22 and harpin were prevented in tobacco deficient in NADPH oxidase (Zhang et al., 2009; Mersmann et al., 2010). In contrast, stomatal closure and ROS production by YEL in Arabidopsis were dependent on SHAM sensitive peroxidases but not NADPH oxidase (Khokon et al., 2010a,b).

Two enzymes, NR and nitric oxides synthase like enzyme (NOA) act as NO sources in guard cells (Gayatri et al., 2013). While the role of NR in contribution to NO-generation is widely accepted, the role of NOA is debated (Neill et al., 2008; Gupta et al., 2011; Agurla et al., 2014). Most of the reports on stomatal closure by elicitors focused on the role of H_2_O_2_ and very few studies were made on the role of NO and its interactions with other signaling components (Melotto et al., 2006; Srivastava et al., 2009; Hao et al., 2010).

There has been great interest to understand the elicitor-induced stomatal closure and the signaling components involved in the process. Most of the work on microbial elicitor-induced stomatal closure was carried out with epidermis of *Nicotiana* (Ye and Murata, 2016). Stomatal closure by harpin, INF1, boehmerin, and Nep1 was reported in *N. benthamiana* (Zhang et al., 2009, 2010, 2012). It would be useful to study the effects of different microbial elicitors on stomatal closure in the same plant, so to assess the common components of signaling pathway. Oligogalacturonic acid and chitosan induced the stomatal closure in tomato, *Commelina communis*, pea, and *Brassica napus* (Lee et al., 1999; Li et al., 2009; Srivastava et al., 2009). Cryptogein and harpin, elicitors from microbial pathogens of tobacco, were shown to induce HR responses and stomatal closure in tobacco (Kadota et al., 2004; Higaki et al., 2007; Zhang et al., 2009, 2012; Kurusu et al., 2013).

*Arabidopsis thaliana* would be an ideal plant for such studies on elicitor triggered stomatal closure. There have been no reports on stomatal closure by cryptogein in *A. thaliana*. Most of the studies with other elicitors were limited to selected signaling components in guard cells of *A. thaliana*, e.g., either ROS or NO, but not with both (**Table 1**). We attempted a comprehensive study on the effects of cryptogein and harpin on stomatal closure as well as key signaling components of guard cells in epidermis of *A. thaliana*, a model plant. We have also examined the kinetics of changes in levels of ROS and NO in guard cells in response to cryptogein and harpin. We extended our studies to evaluate the responses of stomatal guard cells in mutants of *A. thaliana* deficient in signaling components involved in ROS/NO production.

**Table 1:** Reports on ROS or NO production in guard cells during stomatal closure by microbial elicitors in the epidermis of *Arabidopsis thaliana*.

Microbial elicitor	ROS	NO	Reference^∗^
flg22	ROS Not done	Not done NO	Desikan et al., 2008 Melotto et al., 2006
LPS	Not done ROS	NO Not done	Melotto et al., 2006 Desclos-Theveniau et al., 2012
elf26	ROS	Not done	Desikan et al., 2008
Yeast elicitor	ROS	NO	Khokon et al., 2010a
Chitosan	ROS	NO	Khokon et al., 2010b
Cerato-platanin	ROS	Not done	Baccelli et al., 2014
Cryptogein	250 ± 11^∗∗^	200 ± 12^∗∗^	Present report
Harpin_Pss_	185 ± 6^∗∗^	192 ± 7^∗∗^	Present report

*^∗^The first report on the phenomenon.*

*^∗∗^Percentage of control, without elicitor.*

## Materials and Methods

### Plant Materials and Growth Conditions

Seeds of *Arabidopsis thaliana* were sown in a 1:1:1 mixture of vermiculite, perlite and soilrite in plastic disposable containers and kept at 4°C in dark for 3 days and then were transferred to 22–23°C to allow germination. The seedlings were grown in controlled environment growth rooms, under an 8 h light (125–150 mmol m^-2^ s^-1^) and 16 h dark photoperiod, with an average temperature of 22–23°C. The plants were supplied with a nutrient solution (Somerville and Ogren, 1982) or full strength Murashige and Skoog medium (salt mixture obtained from HIMEDIA), on alternate days twice a week. The plants were watered on other days.

### Chemicals

Among the two microbial elicitors, cryptogein was prepared from *Phytophthora cryptogea* (Kadota et al., 2004). Harpin was prepared from *Pseudomonas syringae* pv. *syringae* and was the same as the full length harpin_Pss_ used by Anil and Podile (2012). Both the microbial elicitors were dissolved in milli-Q water, and stock solutions were stored at -20°C. The fluorescent probes of CM-H_2_DCFDA and DAF-FM DA from Invitrogen-Molecular Probes were dissolved in DMSO. cPTIO, L-NAME, and DPI from Calbiochem were dissolved in milli-Q water.

### Bioassay of Stomatal Closure

Leaves from 5- to 6-week-old *A. thaliana* plants were detached and incubated in opening medium (10 mM MES-KOH, pH 6.15 and 50 mM KCl) for 3 h under light. A light intensity of 200 μmol m^-2^ s^-1^ was maintained with the help of a bank of tungsten lamps, and light filtered through a water jacket. The photon flux was measured with a Li-Cor quantum sensor (Li-Cor Instruments Ltd, Lincoln, NE, United States). The temperature was maintained at 25 ± 1°C. After 3 h of light incubation, the leaves were incubated in the medium containing effectors and/or modulators. After treatment for 2 h with the effectors under light, the abaxial epidermis of the leaf was stuck to the cover slip with the help of medical adhesive Telesis V (Premiere Products Inc., Pacoima, CA, United States). The remaining leaf tissue was removed and the stuck epidermis was washed immediately with water. Stomatal apertures were measured with the help of a pre-calibrated research microscope (Olympus CX21) by using NIH image for windows. Approximately 30 stomatal apertures were measured for each experiment and for each treatment.

### Monitoring ROS or NO

The levels of ROS and NO were monitored by using CM-H_2_DCFDA and DAF-FM DA (Excitation 488 nm, Emission 510–550 nm), respectively. The leaves of *A. thaliana* were incubated in an opening medium (10 mM MES-KOH, pH 6.15 and 50 mM KCl) under light for 3 h to allow the opening of the stomata. The abaxial epidermis from leaves were mounted on the cover slips with the help of silicone adhesive and were loaded separately with the 20 μM CM-H_2_DCFDA or 30 μM DAF-FM DA fluorescent probes for 30 min in dark. After incubation, the epidermis of leaves was washed with the buffer to remove the excess dye and was treated with the effectors or modulators.

The fluorescence in the treated guard cells was observed by using confocal laser scanning microscope (Leica, TCS-SP-2, AOBS 4 channel UV and visible, Heidelberg, Germany) for every 5 min up to 30 min, and the images of the guard cells were captured. Average fluorescence of 30 stomata was quantified from the captured images by using NIH Image for Windows (Gonugunta et al., 2008). Averages from three different experiments on different days were presented. The fluorescence of the guard cells without the effectors was taken as control (100%) and the relative fluorescence of the guard cells for different treatment at different time points were calculated and plotted.

### Replication

All the experiments were repeated at least on three different days. The presented data are averages with standard errors.

## Results

### Stomatal Closure by Cryptogein and Harpin, Associated with Rise in ROS or NO of Guard Cells

**FIGURE 1:**
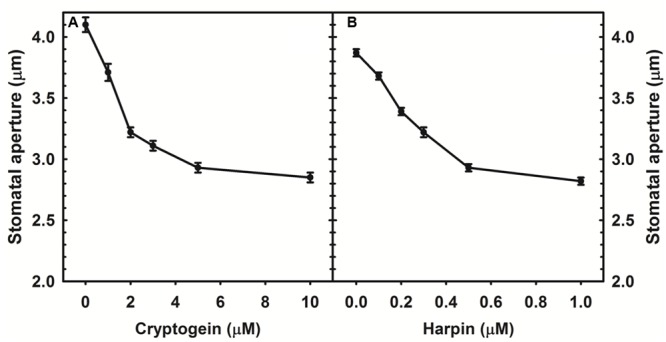
Stomatal closure in response to varying concentrations of microbial elicitors, cryptogein and harpin in *Arabidopsis thaliana*. The two elicitors induced the stomatal closure in a concentration dependant manner and maximum closure was observed at 5 μM of cryptogein **(A)**, 500 nM of harpin **(B)**. Averages of three different experiments from three different days with SE are plotted.

Cryptogein and harpin caused marked stomatal closure in epidermis of *A. thaliana*. Maximum closure already occurred at 5 μM of cryptogein, while harpin induced maximum stomatal closure at 0.5 μM (**Figure 1**).

**FIGURE 2:**
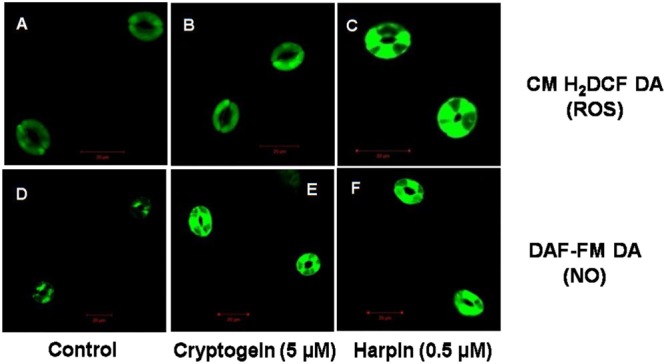
Confocal images of ROS or NO levels in guard cells of *A. thaliana*, treated with cryptogein and harpin. The upper panel **(A–C)** represents the representative confocal images of the guard cells showing ROS levels indicated by CM-H_2_DCFDA fluorescent probe, in response to the microbial elicitors, cryptogein and harpin. The lower panel **(D–F)** represents the confocal images of the NO expressing guard cells by molecular probe DAF-FM DA, in response to the cryptogein and harpin. The levels of ROS or NO were more in the guard cells treated with the two microbial elicitors, were compared to guard cells which were untreated, i.e., control **(A,D)**.

**FIGURE 3:**
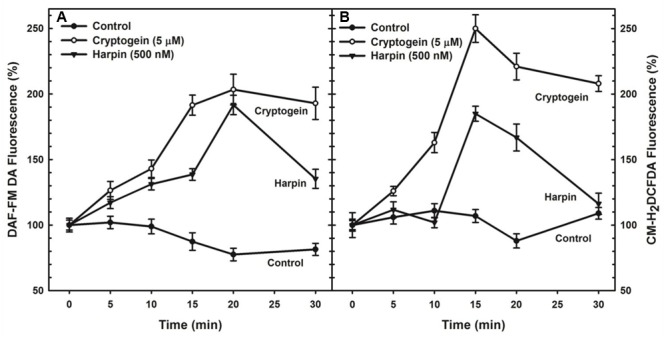
Kinetic studies of NO **(A)** or ROS **(B)** production in guard cells in response to cryptogein and harpin. The levels of NO or ROS raised from 0 to 30 min in the guard cells in response to two microbial elicitors compared with that of untreated guard cells. The fluorescence which is emitted by fluorescent probes, DAF-FM DA or CM-H_2_DCFDA in response to cryptogein and harpin was quantified for every 5 min and plotted in the graph. The data plotted in the graph are the averages of three different experiments on three different days. Further details are in section “Materials and Methods.”

The levels of ROS or NO in guard cells were monitored by using fluorescent probes of CM-H_2_DCFDA and DAF-FM DA, respectively. When treated with the cryptogein, the levels of both ROS and NO increased remarkably, very similar to the effects of harpin (**Figure 2**). Real time monitoring of ROS or NO revealed that maximum accumulation of ROS occurred at 15 min after exposure to cryptogein and harpin. Similarly, levels of NO in the guard cells increased at 20 min after exposure to these two elicitors (**Figure 3**).

### Effect of ROS or NO Modulators on Stomatal Closure and ROS or NO in Guard Cells

**FIGURE 4:**
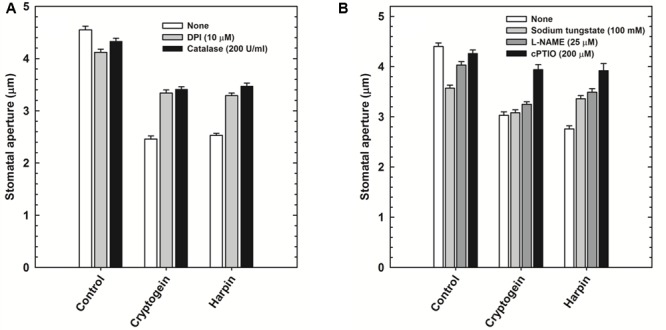
Effect of ROS or NO modulators on stomatal closure by cryptogein and harpin. ROS modulators 10 μM DPI (NADPH oxidase inhibitor) and 200 U/ml catalase (ROS scavenger) relieved the effect of two microbial elicitors, partially **(A)**. Cryptogein and harpin showed the effect on stomatal closure, in the absence of NO modulators, 100 μM sodium tungstate (NR inhibitor), 25 μM L-NAME (NOS inhibitor) and 200 μM cPTIO (NO scavenger). All the NO modulators partially relieved the effect of two microbial elicitors on stomatal closure **(B)**. Averages of three different experiments from three different days with SE are plotted.

The role of ROS or NO during stomatal closure by microbial elicitors, was assessed by using modulators of either ROS or NO. Cryptogein and harpin induced stomatal closure was partially restricted in the presence of ROS modulators, catalase (ROS scavenger), and DPI (NADPH oxidase inhibitor) (**Figure 4A**). Among the modulators of NO, cPTIO (NO scavenger), could reverse the stomatal closure by cryptogein or harpin. In contrast, L-NAME (inhibitor of NOS) and sodium tungstate (NR inhibitor) could restrict the stomatal closure by cryptogein or harpin only to a partial extent (**Figure 4B**).

**FIGURE 5:**
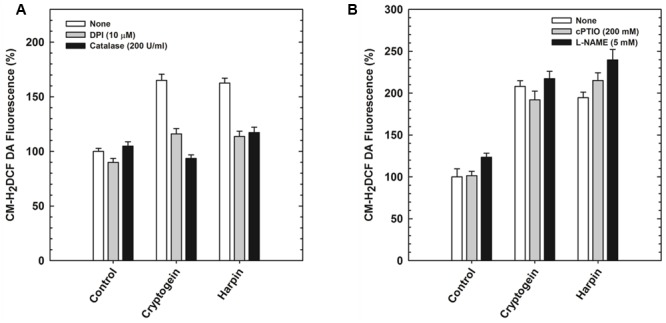
The elevation of ROS levels, (indicated by CM-H_2_DCFDA) by cryptogein or harpin, in the absence or presence of ROS/NO modulators. The levels of ROS in guard cells were more, when treated with cryptogein and harpin, than their respective controls. The extent of increase in ROS by cryptogein or harpin was reduced by ROS modulators (catalase, ROS scavenger and DPI, NADPH oxidase inhibitor) **(A)**. In contrast, elicitor-induced increase was unaffected by NO modulators (cPTIO, NO scavenger and L-NAME, NOS inhibitor) **(B)**. Further details are as in **Figure 3** and “Materials and Methods.”

**FIGURE 6:**
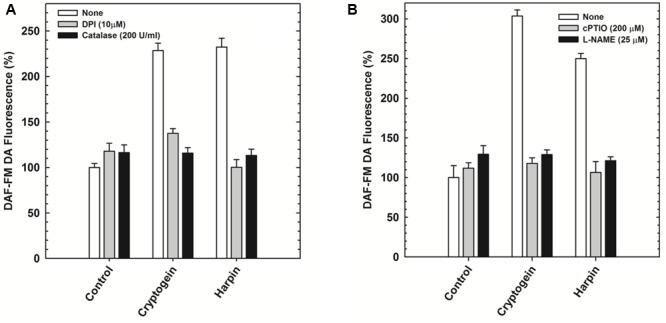
The effect of ROS/NO modulators on the NO production in the guard cells in response to cryptogein or harpin. The levels of NO were indicated by the fluorescence of DAF-FM DA. The ROS modulators, catalase, and DPI restricted the rise of NO in the guard cells when treated with the two microbial elicitors **(A)**. NO modulators, cPTIO and L-NAME relieved the effect of elictors on NO production, where as the rise in NO were observed in the guard cells treated with cryptogein and harpin alone **(B)**. Further details are as in **Figure 3**.

The modulators of ROS (catalase and DPI) restricted the elevation of not only ROS (**Figure 5A**) but also NO (**Figure 6A**) in the guard cells, pretreated with elicitors cryptogein and harpin. In contrast, the modulators of NO (cPTIO and L-NAME) prevented the production of NO (**Figure 6B**), but did not affect the rise in levels of ROS, in the guard cells (**Figure 5B**).

### Elicitor-Induced Stomatal Closure in Arabidopsis Mutants

**FIGURE 7:**
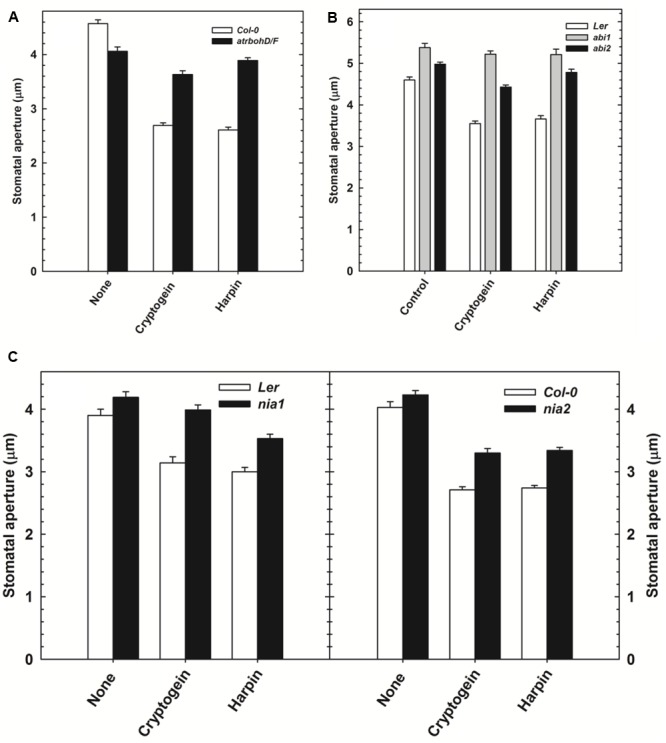
The effect of microbial elicitors cryptogein and harpin on stomatal closure in Arabidopsis mutants. The stomatal closure by elicitors was partial in *atrbohD/F* (NADPH oxidase deficient mutant) plants compared with *Col-*0 (wild type, WT) **(A)**. In contrast, the stomatal closure by elicitors was impaired in the *abi1* (PP2C deficient) and partially impaired in *abi2* **(B)**. The effects of microbial elicitors, cryptogein and harpin were partially relieved in the NR deficient mutants, *nia1* and *nia2* **(C)**. The data plotted in the graph are the averages of three different experiments on three different days. Further details are as in **Figure 1**.

The effect of cryptogein and harpin on stomatal closure was studied in Arabidopsis mutants, deficient in NADPH oxidase (*atrbohD/F*) or NR (*nia1* and *nia2*) or protein phosphatase ABI (*abi1* and *abi2*). The results were compared with wild type, *Col-*0 or *Ler*. The stomatal closure by cryptogein and harpin was partially impaired in *atrbohD/F* mutant plants, compared to wild type (**Figure 7A**). Stomatal closure by cryptogein and harpin was partially relieved in the *nia1* and *nia2* mutants compared with that of their respective wild type *Ler* and *Col*-0 plants (**Figure 7C**). The stomatal closure by cryptogein or harpin was completely reversed in *abi1* mutants (**Figure 7B**), compared to partial impairment in *abi2* mutants.

## Discussion

### *Arabidopsis thaliana* as a Model Plant to Study Stomatal Responses to Microbial Elicitors

The stomatal responses to various biotic and abiotic stress factors have been studied in different plant species including tobacco, *Vicia, Pisum, Brassica napus*, tomato, and *Commelina*. Nevertheless, *A. thaliana* offers as an excellent model compared to the other plant species due to the availability of a large collection of mutants, deficient in the different signaling components. Our article emphasizes the marked stomatal closure by cryptogein and harpin in *A. thaliana* (**Figure 1**). Cryptogein and harpin are well known as elicitors of microbial pathogens of tobacco. Our studies provide for the first time a detailed examination of cryptogein and harpin induced stomatal closure in epidermis of *A. thaliana*. Further, the kinetics and modulation of ROS/NO in guard cells by elicitors are all being reported for the first time in Arabidopsis (**Figures 3, 5, 6**). We suggest that *A. thaliana* offers a good model for comprehensive studies on stomatal signaling events, including the responses to microbial elicitors. Since several mutants of Arabidopsis are available, our article can trigger further interest to use Arabidopsis for elicitor-induced stomatal closure.

### Rise in ROS or NO in Guard Cells by Cryptogein and Harpin

Reactive oxygen species and NO are the important signaling components during stomatal closure by a variety of signals: ABA, MeJA, elicitors like chitosan or even bicarbonate (Kolla and Raghavendra, 2007; Kolla et al., 2007; Gonugunta et al., 2009; Srivastava et al., 2009). The increase in the levels of ROS and NO in the guard cells treated with the cryptogein and harpin (**Figure 2**), confirms their signaling role during closure. There have been reports on increase in ROS as well as NO by cryptogein in epidermal peels of tobacco (Allan and Fluhr, 1997; Foissner et al., 2000) and tobacco BY-2 cells (Kadota et al., 2004; Leborgne-Castel et al., 2008; Stanislas et al., 2009) while inducing cell death and plant defense responses. But the increase in ROS or NO levels in guard cells by cryptogein has so far not been reported.

Harpins from different sources are known to induce production of ROS and NO in Arabidopsis suspension cells (Desikan et al., 1996; Krause and Durner, 2004) and in guard cells of tobacco (Zhang et al., 2009, 2012). Again our article is the first attempt of a comprehensive study on the production of both ROS and NO in guard cells during stomatal closure by harpin in Arabidopsis (**Figure 2**).

### Rise in ROS Occurs before NO Production during Stomatal Closure by Elicitors

Real-time monitoring of fluorescence in guard cells indicated that on exposure to cryptogein and harpin, the levels of ROS rise and reach a peak before that of NO (**Figure 3**). Although the levels of ROS or NO in the guard cells were observed earlier in response to elicitors, such as chitosan, flg22, harpin, boehmerin (Lee et al., 1999; Melotto et al., 2006; Zhang et al., 2009, 2012), studies on kinetics of ROS/NO production are very few. While endorsing the suggestion that ROS production is essential for NO rise in the guard cells during closure by ABA or elicitors (Bright et al., 2006; Srivastava et al., 2009), we conclude that the ROS acts upstream of NO during cryptogein and harpin triggered stomatal closure.

### Interactions of NO and ROS

In guard cells ROS, NO, cytosolic pH, and intracellular calcium are the major points of action, leading to the loss of ions/turgor and subsequent stomatal closure (Agurla and Raghavendra, 2016). Besides their direct effects, there seems to be a strong network of interactions among them. Studies with mutants suggested the upstream action of the ROS to MAP kinases, ABI2 and intracellular Ca^2+^ (Murata et al., 2001; Jammes et al., 2009; Wang et al., 2013). Among the components downstream of ROS are ABI2, NO production, K^+^_in_/SLAC channels (Murata et al., 2001; Zhang et al., 2001; Vahisalu et al., 2008; Srivastava et al., 2009). A rise in NO is considered as the early signaling event in guard cell signaling toward ABA, MJ, bicarbonate (Gayatri et al., 2013). The marked interactions of NO with ROS, phospholipase D and G-protein in guard cells during stomatal closure are known (Gonugunta et al., 2008; Li et al., 2009; Distéfano et al., 2012; Zhang et al., 2012). Further studies on these interactions using elicitors as signals would be extremely interesting.

Another point of interest is the role of mitochondrial AOX. The levels of ROS and NO in mitochondria are minimized by an active AOX (Cvetkovska and Vanlerberghe, 2012; Vanlerberghe, 2013). Unlike cytochrome c oxidase (CytOX), NO resistant AOX plays a major role in preventing excessive NO generation in guard cells, thus regulating the stomatal closure (Cvetkovska et al., 2014; Gupta et al., 2014). Further experiments are warranted to understand the role of AOX in relation to other sources of NO production during stomatal closure by elicitors.

### Cryptogein and Harpins as tools to Induce Stomatal Closure

Cryptogein, a microbial elicitor triggered stomatal closure, and elevated ROS/NO levels in guard cells of *A. thaliana* (**Figures 1, 2**). Besides guard cells, cryptogein has been known to be a potent microbial elicitor in inducing several defense responses, but mostly in tobacco (Montillet et al., 2005; Sawai et al., 2010; Kurusu et al., 2013; Rosnoblet et al., 2017). Similarly, harpin has also been extensively documented for its defense responses again mostly in tobacco (Chen et al., 2008; Boureau et al., 2011; Chang et al., 2016). The marked stomatal closure associated with the rise in ROS/NO of guard cells along with the suitability of *A. thaliana* for these studies opens up an excellent scope for further use of cryptogein and harpin as tools to study stomatal function. The ability of cryptogein and harpin, two elicitors from microbial pathogens of tobacco, to close stomata markedly in Arabidopsis reaffirms the role of stomatal closure as a typical component of innate immunity responses of plants.

## Conclusion

It is quite interesting to note the efficacy of cryptogein and harpin, studied extensively with tobacco, on stomatal closure even in Arabidopsis, a model plant. Our work emphasizes also the ability of these microbial elicitors to induce a marked stomatal closure at very low concentrations, while increasing the levels of ROS and NO in guard cells, *A. thaliana*. We are sure that our work would trigger further use of Arabidopsis to examine the guard cell signal transduction mechanisms during stomatal closure.

## Author Contributions

AR and KK conceptualized the topic and designed the experiments. GG performed most of the experiments. SA and KA conducted some experiments. AR, KK, and AP evaluated the data and drafted the skeleton of manuscript. GG, SA, KK, KA, AP, and AR revised and finalized the manuscript. All the authors read and approved the manuscript.

## Conflict of Interest Statement

The authors declare that the research was conducted in the absence of any commercial or financial relationships that could be construed as a potential conflict of interest. The reviewer RD and handling Editor declared their shared affiliation, and the handling Editor states that the process nevertheless met the standards of a fair and objective review.

## Abbreviations

ABA: abscisic acid
*abi1*: abscisic acid insensitive1
*abi2*: abscisic acid insensitive2
AOX: alternative oxidase
*atrboh*: *Arabidopsis thaliana* respiratory burst oxidase homolog
BY-2: bright yellow-2
CM-H_2_DCF DA: 5-(and-6)-chloro methyl-2′,7′-dichloro dihydro fluorescein diacetate
*Col*: Columbia
cPTIO: 2-phenyl-4,4,5,5-tetramethylimidazoline-1-oxyl3-oxide
DAF-FM DA: 4-amino-5-methyl amino-2′,7′-difluorofluorescein diacetate
DMSO: dimethyl sulfoxide
DPI: diphenylene iodonium chloride
flg22: flagellin 22
HR: hypersensitive response
INF1: *Phytophthora infestans* elicitin, infestin 1
*Ler*: Landsberg *erecta*
L-NAME: *N*-nitro-L-arginine methyl ester
LPS: lipopolysaccharide
MAP kinase: mitogen-activated protein kinase
MeJA: methyl jasmonate
*Nbrboh*,: *Nicotiana benthamiana* respiratory burst oxidase homolog
Nep1: necrosis- and ethylene-inducing peptide 1
*nia1*: nitrate reductase1
*nia2*: nitrate reductase2
NO: nitric oxide
NOA: nitric oxide associated
NOS: nitric oxide synthase
NR: nitrate reductase
OST1: open stomata1
PYR/PYL/RCAR: pyrabactin resistance1/PYR1-like/regulatory component of ABA receptor
RBOH: respiratory burst oxidase homolog
ROS: reactive oxygen species
SHAM: salicylhydroxamic acid
SLAC: slow anion channel
YEL: yeast elicitor.
